# Betaherpesvirus Incidence in Saliva Samples From Patients With Hematological Neoplasms: Frequency, Clinic and Diagnostic Insights

**DOI:** 10.1002/jmv.70770

**Published:** 2026-01-07

**Authors:** Ana Carolina Silva Guimarães, Jéssica Pereira Gonçalves, Nathália de Sousa Pereira, Flávia Freitas de Oliveira Bonfim, Katrini Guidolini Martinelli, Marla Karine Amarante, Sueli Fumie Yamada‐Ogatta, Laura Cinquini Franco, Ligia Carla Faccin Galhardi, Vanessa Salete de Paula

**Affiliations:** ^1^ Molecular Virology and Parasitology Laboratory, Oswaldo Cruz Foundation Rio de Janeiro Rio de Janeiro Brazil; ^2^ Microbiology Department Biological Sciences Center State University of Londrina Londrina Paraná Brazil; ^3^ Social Medicine Department Federal University of Espírito Santo Vitoria Espírito Santo Brazil; ^4^ Pathology, Clinical and Toxicological Analysis Department Health Sciences Center State University of Londrina Londrina Paraná Brazil; ^5^ Clinical Medicine Department Health Sciences Center, Londrina Cancer Hospital State University of Londrina Londrina Paraná Brazil

**Keywords:** betaherpesviruses, hematological neoplasms, qPCR, viral load

## Abstract

Hematological neoplasms (HN) are disorders originating in blood cells that hold significant epidemiological importance. Treatments available for these conditions can induce immunosuppression, and it increases the risk of viral infections and reactivations, mainly by *Human betaherpesviruses* (HCMV, HHV‐6, and HHV‐7). Studies have suggested that these viruses play potential oncogenic role in hematological neoplasms, although results remain inconclusive. This study aimed to evaluate the frequency and viral load of betaherpesviruses in saliva samples from patients with hematological neoplasms, and to explore their relevance to clinicopathological characteristics. In total, 260 saliva samples collected from patients with Hodgkin lymphoma (HL) (*n* = 29), non‐Hodgkin lymphoma (NHL) (*n* = 106), leukemia (*n* = 85) and multiple myeloma (MM) (*n* = 40) were analyzed in multiplex qPCR. The result was compared with control group samples from patients without hematological neoplasm (*n* = 159). HHV‐7 was the most frequently detected betaherpesvirus, identified in 15.8% (41/260) of patients with hematological neoplasms. In comparison, HCMV and HHV‐6 were detected in 12 (4.6%) and 11 (4.2%) patients, respectively. In the control group, HCMV was detected in 2 individuals (1.3%), HHV‐6 in 6 (3.8%), and HHV‐7 in 14 (8.8%). A statistically significant difference in HHV‐7 detection was observed between patients and controls (*p* = 0.005). Additionally, HCMV detection showed a significant difference between patients with HL and MM (*p* = 0.036). The detection of betaherpesviruses, particularly HHV‐7, was more frequent and viral in patients with hematologic malignancies compared to the control group, with statistically significant differences observed. In summary, HHV‐7 was the most frequently detected virus, found in 15.8% of patients versus 8.8% of controls. However, its presence in saliva alone does not confirm disease association. Our findings reinforce the need for longitudinal studies to clarify the potential pathogenic role of HHV‐7 and other betaherpesviruses in hematological neoplasms, and their possible impact on patient outcomes.

## 1 Introduction

Hematological neoplasms (NH) comprise disorders deriving from blood cells, such as myeloid or lymphocytic lineages. They account for 8%–9% of cancer cases diagnosed since 1960 [1, 2]. These disorders encompass several diseases, among them, Hodgkin lymphoma (HL), non‐Hodgkin lymphoma (NHL), leukemia, and multiple myeloma (MM), besides affecting individuals' senescence [3]. The etiology of these malignancies is multifaceted and remains poorly understood. Several risk factors have been identified, such as genetic predisposition, environmental exposure, and lifestyle‐related factors [4].

It is understood that chemotherapy, and other treatments for cancer, can induce immunosuppression in patients with hematological malignancies, and it increases host susceptibility to viral infections or virus reactivation. Accordingly, *Human betaherpesviruses* are acquired early in life, with global seroprevalence close to 90%, which increases due to aging, immunological status, socioeconomic factors, and geographic location [5]. *Human betaherpesviruses* (*Cytomegalovirus humanbeta* 5—HCMV, *Roseolovirus humanbet* 6—HHV‐6, and *Roseolovirus humanbeta* 7—HHV‐7) can infect epithelial cells like monocytes and lymphocytes, which are components of peripheral blood mononuclear cells, and establish latent infections [5]. High viral loads can be detected in blood and serum samples during viral reactivations [5].

Furthermore, saliva is considered a route of transmission of herpesviruses, including betaherpesviruses, and the salivary gland is a particularly permissive site for replication of these viruses [6]. In addition, previous studies published by our group demonstrated the presence of betaherpesviruses in salivary glands tissues and saliva samples, suggesting a possibly tropism for these organs [7]. Other studies in literature recognize saliva samples as biologically relevant and noninvasive specimens for the detection of HCMV, HHV‐6, and HHV‐7 [8, 9, 10].

HCMV is one of the most significant viruses in this family, since its prevalence ranges from 60% to 70% in individuals living in developed countries and reaches ~100% in those leaving developing countries [11]. HCMV is an opportunistic pathogen associated with severe morbidity and mortality cases in immune‐compromised populations, mainly in organ transplant recipients, in individuals with acquired immune deficiency syndrome and in cancer patients (breast, brain, colorectal cancer, and some lymphomas) [12, 13]. Although HCMV is the leading cause of congenital infection, which leads to several birth defect types, such as sensorineural hearing loss and neurological impairments, in children [14]. HCMV infection has been linked to the progression and development of several types of cancer associated with oncogenes like IE, IE2, US28, and UL6 [12]. This virus plays a key role in hematological patients, since acute infection leads to significant morbidity and mortality rates in this population [15].

HHV‐6 was first observed in the blood lymphocytes of adult individuals with lymphoproliferative diseases. This virus is ubiquitous in more than 90% of the human population and its infection takes place in individuals for the first 3 years of life [16]. Studies have shown that HHV‐6 is oncogenic and destructive to autoimmune cells. In addition, its immunomodulatory capacity triggers chronic immunosuppressive and inflammatory pathways [17]. Although the chronic infection remains asymptomatic in the overall population, it is associated with Hodgin's disease, besides other malignancies, in immunocompromised patients [18].

More than 95% of adult humans are persistently infected with HHV‐7 [19]. Overall, the infection caused by it does not lead to clinical complications, although, recently, an increasing number of studies have associated it with severe clinical syndromes, such as transplant complications, neurological impairments, febrile syndromes and dermatological lesions [19]. Despite these complications, some studies reported this virus in patients with hematological malignancies [20, 21, 22, 23]. HHV‐7 reactivation typically happens during immunosuppression periods, in comparison to other herpesviruses, although this virus is often detected in healthy individuals [19].

Although these herpesviruses are often detected in patients with hematological malignancies [15, 17, 24, 25, 26, 27], their association with these health issues remains inconclusive. Furthermore, studies reporting these infections and viral load remain scarce in the literature. Therefore, the aim of the current study was to assess the frequency and viral load of betaherpesviruses (HCMV, HHV‐6, and HHV‐7) in patients diagnosed with hematological malignancies, by analyzing their association with clinicopathological features.

## 2 Methods

### 2.1 Sample Collection and Processing

The current descriptive and retrospective study was approved by the Ethics Committee on Human Research of State University of Londrina (CAAE: 32492720.9.0000.5231).

Samples were collected from September to October 2022, samples were collected at the time of clinical evaluation, typically within 7 days before treatment, 7 days after chemotherapy, and 7 days with completed the treatment. In total, 260 patients from a reference hospital for cancer patients were invited to participate in the study. The control group consisted of 159 individuals without hematological neoplasms. Saliva samples were collected from individuals who accompanied the patients and agreed to participate in the study. All participants signed the informed consent form; legal guardians of underage patients signed the document on their behalf. Inclusion criteria comprised patients diagnosed with HL, NHL, leukemia and MM. On the other hand, exclusion criteria encompassed patients presenting two, or more neoplasms, or autoimmune diseases. Clinicopathological data such as age, sex, clinical diagnosis, survival and death cases, relapse, metastasis, and treatment type were collected from electronic medical records.

Saliva samples were collected from patient oral cavity by using rayon swab (Inlab, São Paulo, Brazil) and placed in cryotubes filled with 2 mL of sterile phosphate‐buffered saline solution (PBS 0.1 M, pH 7.3). Specialized thermal boxes were used to transport these samples to the Basic and Applied Virology Laboratory (LAVIR) at State University of Londrina, where they were stored at −80°C, until processing time.

Biological samples were separated in aliquots and sent by specialized transport to the Molecular Virology and Parasitology Laboratory at Oswaldo Cruz Foundation, Rio de Janeiro, Brazil, where the viral detection process was carried out.

### 2.2 Viral Detection and Viral Load Quantification

Initially, 140 µL of samples were used for nucleic acids extraction by QIAamp DNA Mini kit (Qiagen, Hilden, Germany), according to the manufacturer's recommendations. The extracted samples were stored at −80°C until analysis time.

Quantitative Real‐Time (qPCR) was performed based on using commercial TaqMan Universal PCR Master Mix (Thermo Fischer Scientific, Waltham, MA, USA), to confirm viral detection, as well as to measure viral load through HCMV, HHV‐6, and HHV‐7 target regions U54, U56, and U37, respectively. Multiplex qPCR was performed according to manufacture instruction: the reaction mixture comprising 1 µL 25x PCR Enzyme, 1 µL of each oligonucleotide (3 µM), 1 µL of each probe (0.4 µM), 12.5 µL of 1x PCR Buffer and 2.5 µL of DNA. Oligonucleotides, probes, and synthetic standard curves were previously described by Raposo et al. [28]. Synthetic standard curves ranging from 5 to 5 × 10^8^ copies/µL were used for absolute viral DNA quantification. Ultrapure water and negative samples were used as negative control and positive samples were used as positive control.

To normalize viral DNA quantification, amplification of the human housekeeping gene GAPDH was performed using the TaqMan GAPDH Oligo Mix (20x) (Applied Biosystems, Foster City, CA, USA) in combination with the TaqMan Universal PCR Master Mix, following the manufacturer instructions. Each reaction contained 12.5 µL of Master Mix, 1.25 µL of the GAPDH reagent, 6.25 µL of nuclease‐free water, and 5 µL of DNA. The normalized viral loads were calculated using the ΔCt method (Ct_viral − Ct_GAPDH).

### 2.3 Statistical Analysis

Descriptive statistics applied to the qualitative variables were defined through absolute and relative frequencies, whereas the distribution of quantitative variables was analyzed through the Kolmogorov–Smirnov test. Mean and standard deviation values were used in the analysis due to normal distribution of normal quantitative variables. Inferential statistics used 5% error margin and 95% confidence interval in all the analyses. *χ* ^2^ test was used to compare different neoplasia types presenting viral detection and Betaherpesvirus incidence to clinicopathological parameters set for NHL. Furthermore, ANOVA and Tukey's post hoc test were used to assess differences in viral load among different neoplasia types. Finally, the association between HHV‐7 viral load and clinicopathological parameters was tested through Student's *t*‐test. Statistical analyses were carried out in the Statistical Package for Social Sciences, version 20.0 (SPSS Inc., Chicago, USA).

## 3 Results

### 3.1 Patients' Clinicopathological Features

In total, 53.1% of all 260 patients belonged to the male sex, whereas 46.9% belonged to the female sex. Most patients were White (77.7%) and older than 61 years (45.4%). They were followed by the age group 41–60 years (26.5%) and 13.8% of them were in the age group 19–40 years, on average (Table 1). Most individuals were diagnosed with NHL (40.8%). They were followed by individuals diagnosed with leukemia (32.7%), MM (15.4%), and HL (11.2%). Most of them survived (84.6%). More than 90% of patients did not undergo antiviral therapy. Although, a high rate of HHV‐7 detection was observed in individuals. Clinicopathological features are shown in Table 1, based on hematological neoplasms.

**Table 1:** Data on patients clinicopathological features.

Parameters	*N* = 260	%
Sex		
Male	138	53.1
Female	122	46.9
Age at diagnosis time (years)		
Mean (SD)	52	23
Median (IQR)	58	58
0–10	17	6.5
11–18	20	7.7
19–40	36	13.8
41–60	69	26.5
> 61	118	45.4
Ethnicity		
White	202	77.7
Black and Brown	58	22.3
Hematological neoplasm		
Leukemia	85	32.7
Myeloma	40	15.4
Hodgkin lymphoma	29	11.2
Non‐Hodgkin lymphoma	106	40.8
Clinical evaluation		
Before treatment	133	51.2
After chemotherapy	80	30.8
Completed treatment	47	18.0
Antiviral treatment		
Without antiviral	237	91.2
With antiviral	23	8.8
Metastasis		
Presence	254	97.7
Absence	6	2.3
Relapse		
Without relapse	220	84.6
With relapse	40	15.4
Remission		
Without remission	163	62.7
With remission	97	37.3
Viral detection		**%**
HCMV	12	4.6
HHV‐6	11	4.2
HHV‐7	41	15.8
Coinfections		
HHV‐6/HCMV	0	0.0
HHV‐7/HCMV	7	2.7
HHV‐7/HHV‐6	5	1.9

### 3.2 Detection of Betaherpesviruses and Their Association With Patients Clinicopathological Features

Median values about betaherpesviruses presence and absence and association with clinicopathological features are shown in Table 2. A rate of 50.0% of viral positivity was observed in NHL samples. Most patients before treatment presented a rate of 44.2% of positivity for HHV‐7. Statistical significance was observed in those after chemotherapy (*p* = 0.049).

**Table 2:** Betaherpesviruses detection and patients clinicopathological features.

**Parameters**	HCMV	HHV‐6	HHV‐7	*χ* ^2^
Clinical evaluation				0.049
Before treatment	8 (15.4)	5 (9.6)	23 (44.2)	
After chemotherapy	3 (5.8)	5 (9.6)	17 (32.7)	
Completed treatment	1 (1.9)	1 (1.9)	0	
Antiviral treatment				0.421
With antiviral	1 (1.9)	1 (1.9)	4 (7.7)	
Without antiviral	11 (21.1)	10 (19.2)	36 (69.2)	
Metastasis				1.00
Presence of metastasis	0	0	1 (1.9)	
Absence of metastasis	12 (23.1)	11 (21.1)	39 (75.0)	
Relapse				0.086
With relapse	0	1 (1.9)	3 (5.8)	
Without relapse	11 (21.1)	10 (19.2)	37 (71.1)	
Remission				0.847
With remission	6 (11.5)	3 (5.8)	11 (21.1)	
Without remission	5 (9.6)	6 (11.5)	39 (75.0)	

### 3.3 HCMV, HHV‐6, and HHV‐7 Detection in Patients With Hematological Neoplasm

If one takes into consideration the hematological neoplasm subgroups, HHV‐7 was the most detected betaherpesvirus, 15.8% of cases (41/260). Statistical significance was observed through HCMV detection between HL and MM (*p* = 0.036). HHV‐7 detection was statistically significant in all hematological neoplasm cases (*p* = 0.005) (Table 3). In relation to the control group, 2 (1.25%) individuals were positive for HCMV, 6 (3.77%) for HHV‐6, and 14 (8.80%) HHV‐7.

**Table 3:** Viral detection and coinfection rates in the assessed patients.

		Hematological neoplasms subgroups				
	Total	Hodgkin lymphoma *N* (%)	Non‐Hodgkin lymphoma *N* (%)	Leukemia *N* (%)	Myeloma *N* (%)	
	(*n* = 260)	*N* = 29	*N* = 106	*N* = 85	*N* = 40	*χ* ^2^
HCMV						0.036
Negative	248 (95.4)	26 (89.7)	100 (94.3)	82 (96.5)	40 (100)	
Positive	12 (10.0)	3 (10.3)	6 (5.7)	3 (3.5)	0 (0.0)	
HHV‐6						0.966
Negative	249 (95.8)	28 (96.6)	101 (95.3)	82 (96.5)	38 (95.0)	
Positive	11 (4.2)	1 (3.4)	5 (4.7)	3 (3.5)	2 (5.0)	
HHV‐7						0.005
Negative	219 (84.2)	23 (79,3)	84 (79.2)	72 (84,7)	40 (100)	
Positive	41 (15.8)	6 (20.7)	22 (20.8)	13 (15.3)	0 (0.0)	
Coinfections		Coinfections [*n* (%)]				
HHV‐7/HCMV	7 (58.3)	2 (6.9)	3 (2.8)	2 (2.4)	0 (0.0)	0.113
HHV‐6/HHV‐7	5 (41.7)	4 (75.0)	0 (0.0)	1 (1.1)	0 (0.0)	
HHV‐6/HCMV	0 (0.0)	0 (0.0)	0 (0.0)	0 (0.0)	0 (0.0)	

### 3.4 Human Betaherpesvirus Viral Load

**Figure 1:**
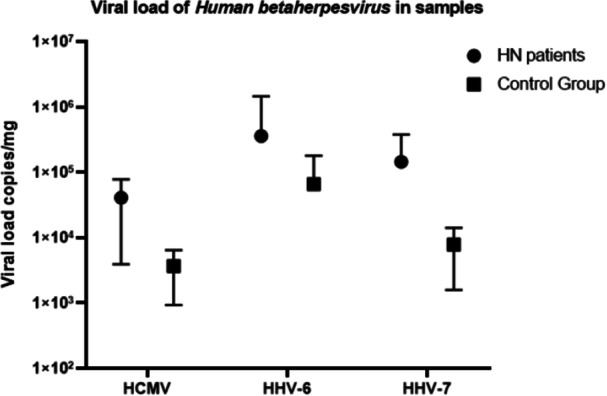
Viral load of betaherpesviruses in the analyzed samples. The black bar circle symbol refers to HN patients, and the black square refers to control group.

HHV‐6 recorded a viral load of 3.59 × 10^5^ in the total number of analyzed samples, whereas HHV‐7 recorded 1.44 × 10^5^, which was the highest viral load observed in these samples in comparison to HCMV (4.08 × 10^4^) (Figure 1).

**Figure 2:**
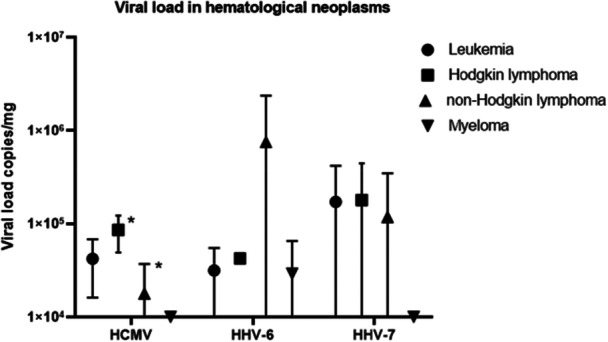
Viral load in hematological neoplasms. *Turkey test has shown a significant difference in HCMV's mean viral load between HL and NHL cases (*p* = 0.014).

Based on the analysis applied to hematological neoplasms, HHV‐6 recorded the highest viral load in patients with NHL, whereas HHV‐7 presented the highest viral frequency in individuals with HL and Leukemia (Figure 2). Statistical significance was observed between HCMV's mean viral load between HL and NHL cases (*p* = 0.014).

The GAPDH quantification cycle (Ct) ranged between 34.341 and 39.515, which is within the expected range for low‐input biological samples such as oral swabs. These Ct values, while high, were consistently detected across all samples, supporting the use of GAPDH as a reliable reference for normalization in this context. The GAPDH was detected in all samples, and the integrity of saliva samples was ensured.

### 3.5 HHV‐7 Viral Load and Patients' Clinicopathological Features

Comparative analysis between viral load and patients with clinicopathological features was carried out because HHV‐7 was the most detected virus. Statistical significance and high viral load were observed in patients after chemotherapy for hematological neoplasms (2.63 × 10^5^) (*p* = 0.020) and in those presenting metastasis (8.14 × 10^5^) (*p* = 0.002).

## 4 Discussion

The current study focused on detecting *Human betaherpesvirus* and on calculating the viral load of these viruses in saliva samples collected from patients with hematological neoplasms, as well as on correlating them with clinicopathological features presented by these individuals. There was a high HHV‐7 detection rate with a high viral HHV‐6 load in these samples. Some clinicopathological features, such as metastasis, were associated with high HHV‐7 viral load. Although Human Herpesvirus 7 (HHV‐7) is a ubiquitous virus capable of establishing latency in host cells, current evidence does not support a direct role in oncogenesis. Unlike other herpesviruses such as the Epstein–Barr virus (EBV) and Kaposi's sarcoma‐associated herpesvirus (HHV‐8), which are well‐established oncogenic agents, HHV‐7 has not been classified as carcinogenic. However, some studies suggest that HHV‐7 may influence cellular processes related to tumorigenesis, including cell cycle regulation, immune evasion, cell‐to‐cell spread, cytokine modulation, and interference with DNA mismatch repair pathways [19, 27, 29]. While HHV‐7 DNA has been detected in certain tumor tissues, these findings remain inconclusive and require further investigation. To date, definitive molecular mechanisms need to be investigated linking HHV‐7 to cellular transformation or cancer development.

High HHV‐7 detection rate was recorded for saliva samples collected from patients and they presented statistically significant with leukemia, myeloma, HL, and NHL. Other studies had previously reported betaherpesviruses in saliva samples collected from renal patients [28] and from neoplastic and non‐neoplastic salivary gland tissues [7], and it evidenced viral activity in this organ and shedding of viral particles in these patients' saliva. Rizk and Darwish conducted a study with 60 children diagnosed with acute lymphoblastic leukemia who were tested for EBV, HHV‐6, and HHV‐7 by qPCR. They reported high HHV‐7 incidence in these patients, a fact that corroborated findings in the current study [30]. Another study detected HHV‐7 in bone marrow and peripheral blood samples collected from children with acute lymphoblastic leukemia [20]. However, none of these studies has evidenced an association between HHV‐7 infection and this disease.

NHL was the most common neoplasm observed in samples collected from both women and men in the age group 41–61 years, in White individuals and in those presenting relapse rates. NHL is the most common hematological malignancy worldwide; it accounts for ~3% of cancer diagnoses and associated death cases [31]. In 2020, ~544 000 new NHL cases and 260 000 related death cases were recorded at global scale. This finding indicates that NHL accounted for ~2.6% of all cancer‐related death cases in that year, worldwide [32]. Individuals with aggressive NHL, mainly children and teenagers, presented relapse/refractory disease with accurate rates < 30% [33].

High leukemia incidence rates were observed in patients in the age group 0–19 years. On the other hand, it was mostly diagnosed in black and brown individuals at the age of 41 who recorded high metastasis rates. Recent data have indicated ~643 579 new leukemia cases in 2019 and 334 592 death cases, worldwide. This finding reflected an increase in the number of leukemia diagnosis and death‐related cases in comparison to previous years [34].

Another aspect observed in the current study was that HHV‐6 recorded the highest viral load in the analyzed samples in comparison to HCMV and HHV‐7 in patients with leukemia. Based on the literature, HHV‐6 is often asymptomatic or associated with febrile illness known as exanthema subitum, and it has potential to reactivate during immunosuppression [35]. High viral loads can lead to complications in some cases; some studies previously reported the association of high HHV‐6 viral loads with delayed platelet engraftment and encephalitis [36, 37]. Another study reported that high HHV‐6 viral load increases mortality risk in patients undergoing allogeneic hematopoietic stem cell transplant [38]. These findings underscore the clinical significance of HHV‐6 reactivation in immunosuppressed patients, besides emphasizing the need to carefully monitor this population. Furthermore, HHV‐6 recorded the highest viral load in the NHL patients assessed in the present study. Kiani and colleagues tested 44 HL and NHL tissue samples for HHV‐6A/B; all NHL samples tested positive for HHV‐6A [39].

The current study observed high HHV‐7 viral load in patients with metastasis and a high viral frequency found in patients after chemotherapy. HHV‐7 is linked to several lymphoproliferative cancer types, as well as to pediatric lymphoma, NHL, HL, acute leukemia, basal cell carcinoma, and glioma [40]. HHV‐7 can infect both primary CD4+ T lymphocytes and the SupT1 lymphoblastoid T‐cell line [40]. This factor can contribute to cancer development due to cell accumulation in Gap 2/mitosis phase, as well as to polyploidy and increased cell size [40]. However, literature lacks evidence of HHV‐7 infection associated with hematological malignancies, such as leukemia. Previous study reported high HHV‐7 rate in HL and NHL biopsies, but its biological significance remains unclear [22].

Although, the detection of HHV‐7 in saliva should be interpreted with caution, particularly in immunocompromised patients. While the presence of the virus may indicate viral reactivation, its high prevalence in healthy individuals and across various clinical conditions suggests that detection alone is insufficient to establish a direct pathological association with hematological disorders [41]. Recent studies have shown that HHV‐7 is commonly found in the saliva of healthy individuals, and its frequency increases in immunocompromised populations, such as HIV‐infected patients [6]. In our study, the frequency of HHV‐7 in saliva was 15.8% (41/260) and in the control group 8.80% (14/159). Longitudinal studies including immunocompetent controls are warranted to better elucidate the potential pathogenic role of HHV‐7.

HCMV is also not classified as oncogenic virus, although studies have shown the association of this virus with some human cancer types [21, 42, 43, 44]. HCMV can persist as latent infection on the host for a lifelong time and leads to a whole series of disorders [44]. HCMV associations with HL and NHL have been reported. Mehravan and colleagues reported the prevalence of HCMV latent infection in histological tissue collected from patients with HL and NHL. They detected UL138 protein and HCMV's IE1 replication gene in the analyzed tissue [44]. Bogner and Pecher conducted a study with patients with MM and tested their humoral response for HCMV. They used a recombinant immunoblot test and recorded 80% IgG immune response to HCMV; this finding has evidenced viral infection in the assessed patients [45]. The HCMV viral load observed was statistically significant in patients with HL and NHL. On the other hand, HCMV viral detection was statistically significant in individuals with HL and MM.

Diagnostic insights into HHV‐6 and HHV‐7 in hematologic malignancy cases have emphasized the importance of detecting viruses capable of reactivating. According to recent studies, high HHV‐6 and HHV‐7 levels were associated with clinical complications and with increased posttransplant mortality rates, a fact that reinforces the need to adopt diagnostic approaches capable of combining accurate viral load quantification to patients detailed clinical assessment. These insights not only improve scientific understanding about viral behavior in hematologic malignancies but also pave the way for personalized therapeutic strategies. Although, this study has some limitations that should be considered when interpreting the findings. Saliva samples were collected from patients at different stages of hematological neoplasm treatment, which may have influenced viral detection due to variations in immune status and treatment‐related immunosuppression. Despite this heterogeneity, the detection of HCMV, HHV‐6, and HHV‐7 in patients with hematological malignancies is relevant for understanding viral reactivation and its potential implications for disease progression and clinical management. Notably, HHV‐7 was the most frequently detected virus, observed in 15.8% of patients and 8.8% of controls. However, its presence in saliva alone is not sufficient to establish a causal relationship with disease. Few studies have evidenced association between HHV‐7 viral detection and viral load quantification in hematological neoplasm cases. This factor can be explained by the fact that this virus is not seen as oncogenic; consequently, it is the least investigated virus in this context. Moreover, patients undergoing chemotherapy require attention regarding the reactivation of viruses, such as HHV‐6, otherwise it can lead to a poor prognosis. These findings underscore the need for longitudinal studies to further elucidate the pathogenic potential of HHV‐7 and other betaherpesviruses in hematologic disorders and to evaluate their possible impact on treatment outcomes.

In summary, HHV‐7 was the most frequently detected virus found in 15.8% of patients versus 8.8% of controls. However, its presence in saliva alone does not confirm disease association. Our findings reinforce the need for longitudinal studies to clarify the potential pathogenic role of HHV‐7 and other betaherpesviruses in hematological neoplasms, and their possible impact on patient outcomes.

## Author Contributions

All attributions were equally divided among the authors, as well as the two herein carried out research activities and the preparation of the present study.

## Ethics Statement

The current descriptive and retrospective study was approved by the Ethics Committee on Human Research of State University of Londrina (CAAE: 32492720.9.0000.5231).

## Consent

Written informed consent was obtained from all individual participants included in the study.

## Conflicts of Interest

The authors declare no conflicts of interest.

## Data Availability

The authors have nothing to report.
